# About half of Ethiopian midwifery professionals reported being dissatisfied with their jobs: A systematic review and meta-analysis

**DOI:** 10.1016/j.eurox.2023.100277

**Published:** 2023-12-28

**Authors:** Dagne Deresa Dinagde, Shambel Negesa marami, Gizu Tola Feyisa, Bekem Dibaba Degefa

**Affiliations:** Departments of Midwifery, College of Health Sciences, Mattu University, Mettu, Ethiopia

**Keywords:** Job satisfaction, Midwives, Job turnover, Ethiopia

## Abstract

**Background:**

Increasing well qualified health professionals is a part of sustainable development goal to specially to decrease maternal mortality below 70 per 100,000 deaths. Contrarily, The Nursing and midwifery councils (NMC) expect that 36% of healthcare workers, especially midwives, are leaving their jobs due to high turnover rates and job unhappiness worldwide.

**Methods:**

Studies were rigorously searched utilizing international databases from PubMed, Google Scholar, Cochrane Library, and Embase. Using the New Castle Ottawa scale for a cross-sectional study design, the quality of the articles that were searched was evaluated. The systemic review was conducted using the random effect approach, and statistical analysis was done using STATA version 17 software for the window. The Preferred Reporting Item for Systematic Review and Meta-Analyses (PRISMA) guideline was followed for reporting results.

**Results:**

A total of nine observational cross-sectional studies were included in this review. The pooled level of job satisfaction among midwives in Ethiopia was 52.2% (95% CI =41.7, 62.9). The pooled odds ratio showed that a significant positive association was found between midwives’ job satisfaction and studied variables. Male midwife (OR = 0.45; 95% CI: 0.04, 0.87), fair supervision (OR = 2.03; 95%CI: 1.58–1), workload (OR = 1.72; 95%CI: 1.102–2.43) and motivation (OR = 1.64; 95%CI: 1.02–2.25) were strongly associated with job satisfaction.

**Conclusion:**

Evidence suggested that motivating employees, providing fair supervision, fair workloads, and fostering positive relationships with managers are all crucial tactics for retaining and enhancing the satisfaction of health professionals at health care facilities in Ethiopian.

## Background

National, regional, and global initiatives, such as the WHO HRH 2030 Strategy, are being made to address the problems related to human resources for health and ensure that all people have equitable access to highly qualified and motivated healthcare providers, who are an essential component of a well-functioning health system [1]. For instance, Ethiopia's HRH strategy plan for 2016–2025 states that in 2016, there were 8200 people for every midwife (the international benchmark is 1 for 5000). Despite advances, more work has to be done to increase pre-service education and training programs for health professionals in order to reach the minimal threshold of 2.3 health workers per 1000 people as advised by the World Health Organization (2006). Reducing mortality and morbidity in mother and child health, especially in Ethiopia, requires increasing and improving the capacity of midwives [2].

Increasing well qualified health professionals is a part of sustainable development goal to specially to decrease maternal mortality below 70 per 100,000 deaths [3]. Therefore, increasing the number of health professionals with expertise in pregnancy and associated fields (i.e. midwives) is essential to achieving the aim. According to Stamps (1997), job satisfaction is "the degree to which employees like their jobs" [4]. One of the most crucial elements affecting the effectiveness and productivity of human resources is job satisfaction [5].

The level of job satisfaction among midwives is a complex issue affected by numerous factors. Compensation (benefits), recognition by management, adequate supportive supervision, good reward and recognition, high normative commitment and opportunity for development, were associated with job satisfaction. A unit increase in salary and incentives and recognition by management scores resulted in best job happiness [6], [7]. Job unhappiness is widely identified as the main cause of midwives' high absenteeism and turnover rates, which endanger the ability of a healthcare organization to offer high-quality treatment by hindering their efficacy and efficiency. As a results about 36% of midwives leave their job globally every year [8]. The UNFPA has urged for changes to the workplace to help recruit and keep midwives in the profession given the significance of midwives in all facets of sexual, reproductive, maternal, infant, and adolescent health care [9].

Numerous research carried out in Ethiopia revealed that there are still active role disputes in the labor wards of many hospitals, making the workplace uncomfortable for midwives. The concepts of midwifery (births as normal happenings) and medicine (births as events requiring specialist medical support) led to role disputes between obstetricians and midwives. Role conflicts at work involving topics like leadership, decision-making, and support for the expectant mother were caused by these overarching divergent principles. Collaboration between different professions was made more challenging by the ambiguity around professional responsibilities in the team surrounding laboring women [10], [11], [12], [13].

To sum up, although midwives work in strained environments, their work is meaningful, and professional proud that saves priceless maternal life. Thus, it is of utmost importance to estimate the national level of stressfulness and dissatisfaction. However, across the country, the results of current studies are variable and equivocal, which makes evidence-based solutions difficult. This systematic review and meta-analysis will therefore aim to determine the level of job satisfaction among midwives to awaken policy makers to improve midwives’ attrition, and intention to leave, and reduce midwives displacement in Ethiopia.

## Methods

### Study design and setting

This systematic review and meta-analysis were conducted to assess the pooled level of job satisfaction and contributing factors among midwifery professionals in Ethiopia. Ethiopia is a landlocked country found in the Horn of Africa with an estimated population of above 121 million. It shares the boundary with Eritrea to the north, Djibouti to the northeast, Somalia to the east, Kenya to the south, South Sudan to the west, and Sudan to the northwest [14].

### Reporting

The results of this review were reported based on the Preferred Reporting Items for Systematic Review and Meta-Analysis statement (PRISMA) guideline [15]. However, it was not registered on the prospective registration of systematic review and meta-analysis (PROSPERO), which is addressed in Limitations of the Study.

### Search strategies

A comprehensive search of studies was conducted using distinct databases such as PubMed, DOJA, Embase, Science Direct, Cochrane Library, African Journals Online, Google Scholar, and Web of Science. In the beginning, studies were comprehensively searched using the full title (“Level of job satisfaction and its determinants among midwives working at health facilities in Ethiopia:”) and keywords (“Level of job satisfaction,” “midwives,” “job satisfaction,” “determinant factors,” “job turnover,” “associated factors,” and “in Ethiopia”). Boolean operators “OR” or “AND” were used in combination or independently to connect these keywords and to establish the search terms. Besides, reference lists of all included studies were considered to find other missed studies. Additionally, institutional repository from Jimma University was used to search important literatures. A literature search was conducted from July 10 to August 8, 2023.

The Population, Exposure, Comparison, and Outcomes statement was also used in this review; Population: (midwifery professionals who are working in health care settings) in Ethiopia; Exposure: determinants of job satisfaction and burnout such as work experience and socio-demographic characteristics; Comparison: reported reference group in each respective study; and Outcome: job satisfaction among midwives.

## Eligibility criteria

### Inclusion criteria

To establish the inclusion and exclusion criteria for this systematic review and meta-analysis, the authors used the PICO technique, which mainly involves Condition, Context, and Population (CoCoPop) questions for prevalence studies.

*-Study area*: Studies conducted in Ethiopia.

*-Study design*: All observational studies that report either the proportion or level or magnitude and associated or determinants factors or predictors of midwives job satisfaction.

-Studies with the outcome of interest published in English language for the sake of clarity, and simplicity of interpretations were included.

*Publication condition*: Both published and unpublished studies.

*-Time:* No limitation was made for the time of publication and all studies conducted in Ethiopia were included.

### Exclusion criteria

Studies with a different result of interest and qualitative research that couldn't give quantitative support for the pooled estimate were removed. Besides this, studies with different operational definitions and measurements of midwives’ job satisfaction were excluded from this systematic review and meta-analysis.

### Data extraction

Three authors (GT, BD and SHN) separately extracted the data from the included studies using a piloted data extraction Microsoft Excel spreadsheet. The spreadsheet includes the first author's name, publication year, study year, study design, study area, study setup, sample size and the proportion/level of job satisfaction among midwifery professionals. When there was any discrepancy between the reviewers, the differences were resolved through discussion and reevaluation of each study.

### Data quality assessment

Using a standardized data appraisal format adapted from the Newcastle-Ottawa Scale (NOS), we had carried out a critical appraisal of the research evidence to evaluate the methodological quality of a study and ascertain the extent to which a study has addressed the possibility of bias in its design, conduct, and analysis. The tool uses a star grading system and includes three key components. The first component, which has a maximum rating of five stars, takes into account the veracity of the research group selection process. The comparability of the groups with a potential for two stars is the subject of the tool's second section. The final component of the grading system concentrates on the determination of each original study's exposure or outcome, with the potential for three stars to be evaluated. Then the quality appraisal of included studies was evaluated by two authors (BD and GT) independently and any discrepancy was resolved by other authors (DD and SHN). Articles with a NOS score of ≥ 5 stars out of 10 were considered as high quality [16] for the purposes of our work (Table 1).

**Table 1:** Summary of characteristics of included studies.

Author’ name and year of publication	Year of study	Study area	Region	Study setting	Study design	Sample size	Magnitude	Quality
Muleneh et al.,2022[10]	2020	4 developing region	Gambella, Somali, B/Gumuz & Affar	IB	CS	107	48 (45%)	Low risk
Temesgen et al., 2018[18]	2016	Western Amhara	Amhara	IB	CS	76	16 (21.1%)	moderate risk
Bekru et al., 2017[11]	2015	Addis Ababa	Addis Ababa	IB	CS	221	117 (53%)	low risk
Meselu et al., 2020[25]	2014	Central zone Tigray	Tigray	IB	CS	140	61 (43.6%)	Low risk
Kebede et al.,2023[21]	2022	Jimma zone	Oromia	IB	CS	121	65 (53.7%)	Low risk
Fenta et al., 2023[21]	2022	Northwest Amhara	Amhara	IB	CS	636	284 (44.7%)	Low risk
Mekuria et al.,[22]	2012	West shoa zone	Oromia	IB	CS	24	12 (50%)	moderate risk
Abajebal et al.,[23]	2014	West Hararge	Oromia	IB	CS	98	77 (78.6%)	Low risk
Gesesew et al., 2016[24]	2014	Jimma zone	Oromia	IB	CS	29	24 (94.4%)	moderate risk

IB-Institutional Based, CS- Cross sectional

### Measurement of the outcome of interest

The major outcome of this systematic review and meta-analysis was the level/magnitude of job satisfaction among midwifery professionals in Ethiopia. The secondary outcome variable was determinant factors of job satisfaction which were estimated using a pooled adjusted odds ratio (OR) with 95% confidence intervals (CIs). Health professionals (midwives) were considered to be satisfied with their job when they score above the mean of job satisfaction subsidiary scale.

### Statistical methods and analysis

The extracted data from Microsoft Excel spreadsheet were imported to STATA™ 17 statistical software for analysis. To calculate pooled estimation of overall job satisfaction MetaAnalyst beta 3.3 version of software was used. The percentage of total variation among studies that is attributable to heterogeneity rather than chance is expressed by the I^2^ statistic, which we calculated to assess the degree of study heterogeneity. A score of 30% to 60% on the I^2^ statistic may indicate moderate heterogeneity, a value of 50% to 90% may indicate substantial heterogeneity, and a value of 75% to 100% may indicate significant heterogeneity [17]. Therefore, a random effects model using the DerSimonian–Laird method was used to estimate the pooled level of job satisfaction. The OR and 95% CIs were constructed from the included studies.

*Subgroup analyses and heterogeneity*: To identify possible causes of heterogeneity amongst the included studies, subgroup analyses based on geographic regions and studies’ quality were conducted.

### Publication bias and heterogeneity

The non-symmetrically distributed inverted funnel plot of this study indicates publication bias **(**Fig. 3**).** The overall heterogeneity of job satisfaction included was Ι^2^ = 93.3%, with P < 0.001 by use of the random effect model to adjust observed variability (look at Fig. 3).

**Fig. 1:**
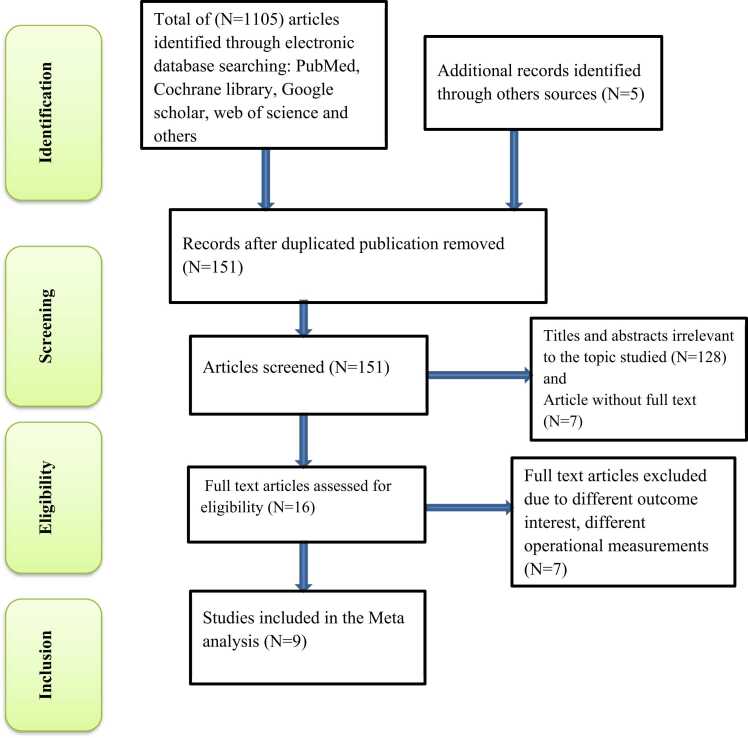
-Flow chart diagram describing selection of studies included in the systematic review and meta-analysis using PRISMA checklist, 2023.

**Fig. 2:**
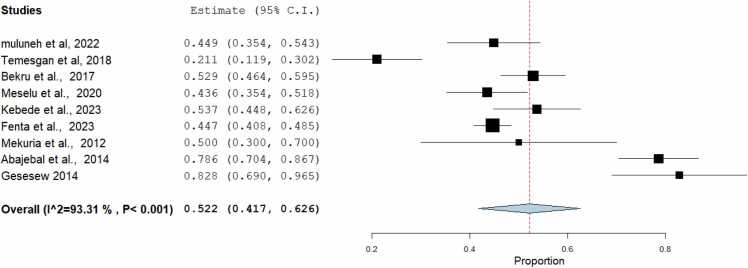
-Overall pooled estimates of job satisfaction among midwives in Ethiopia, 2023.

**Fig. 3:**
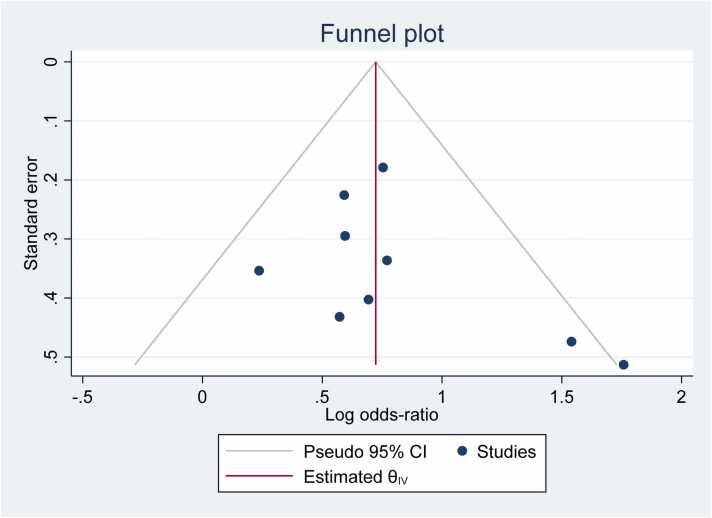
funnel plot showing the study’s publication bias, 2023.

*Sensitivity analysis:* A random effects model was used for sensitivity analysis to determine the impact of one study on the overall level of job satisfaction.

## Results

A total of 1110 published and unpublished studies were found after a search of studies through various worldwide databases and the institutional repository of Ethiopian institutions. The obtained studies were screened using the endnote reference manager, and duplicate studies were eliminated. Thus, 151 articles were enrolled for the abstract and title screen, 959 articles were eliminated from consideration due to duplications and irrelevancies, and 16 complete papers were retrieved. Finally, 9 studies that satisfied the inclusion criteria were included in this study analysis (Fig. 1).

### Characteristics of selected studies

As described below (Table 1**)**, all of these papers were published between 2012 and 2023. In the current systematic review and meta-analysis, 1452 midwives participated in estimating the pooled level of job satisfaction among midwives working at various health institutions in Ethiopia. All study designs included in this study were cross-sectional study. The magnitude /level of job satisfaction among midwives were ranged from 21.5% to 94.4%. Seven geographical regions including the nation's capital city of Addis Ababa were represented in this meta-analysis; two studies were from the Amhara region [18], [19], [20], four studies were from Oromia [21], [22], [23], [24], one study from Tigray region [25], one study from Addis Ababa city [11], one other study was from developing regions; Gambella, Affar, Benishangul Gumuz and Somali [10], [26].

### Level of job satisfaction

The overall pooled level of job satisfaction among midwives working at health facilities in Ethiopia was found to be 52.2% (95% CI =41.7, 62.90) (Fig. 2 ). Study heterogeneity was examined using the I^2^ test, and the results revealed that there was a significant degree of variation between the studies (I^2^ =93.3%, P value < 0.001). The DerSimonian-Laird approach was used in a random effects model to calculate the pooled level of job satisfaction (Fig. 2 ).

**Publication Bias**.

### Subgroup analysis

Based on the geographical location of the regions, the subgroup analyses revealed that Oromia had the highest pooled estimate of job satisfaction at 67% (95%CI: 50.7–83.2), followed by the Amhara region at 33.2% (95%CI: 11–55.4), the developing regions at 42.8% (95%CI: 33.1–52.5), and the other regions at 47.7 (95%CI: 41.1–54.2). Subgroup meta-analysis of the level of job satisfaction by quality of included studies indicated that the level of job satisfaction was 53% (95%CI: 43.1–62.9) and 51% (95%CI: 29.2–76.4) among low quality studies (Table 2).

**Table 2:** Sub-group analysis of studies included in meta-analysis on factors affecting job satisfaction in Ethiopia, 2023.

Subgroup	Random effects of (95%CI)	Heterogeneity (I^2^)
Regions		
Oromia	67% (95%CI: 50.7–83.2)	87.47%
Amhara	33.2% (95%CI: 11–55.4)	95.3%
Others*	47.7 (95%CI: 41.4–54.2).	45.88%
Quality of study		
low risk	53% (95%CI: 43.1–62.9)	91.63%
moderate risk	51% (95%CI: 29.2–76.4)	94.94%

Others; - Addis Ababa city and Tigray region

## Determinants of job satisfaction

Due to inconsistent categorization (grouping) of the exposures in relation to the outcome (job satisfaction), some of the components related to job satisfaction in this review were quantitatively pooled while others were not. From the included studies, 20 variables were taken to determine major determinants of job satisfaction. Finally, 4 variables (fairness of supervision, motivation, sex of respondents and workload) were identified as significant determinants of job satisfaction.

**Fig. 4:**
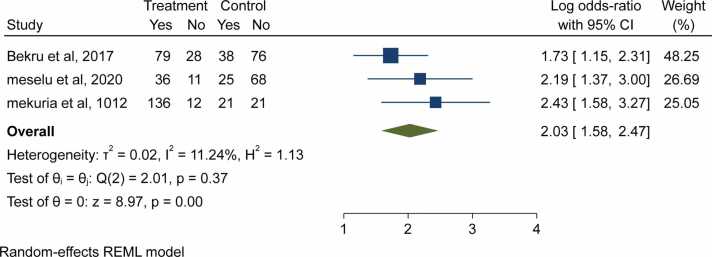
- Forest plot for the association between fair supervision and job satisfaction among midwives in Ethiopia, 2023.

According to three studies, people who agreed with the fairness of supervision were more likely than their counterparts to be satisfied with their jobs. The other studies that were still included in the study pool revealed that job satisfaction and supervision had no association. Thus, the pooled data showed that midwives who working in facilities with fair monitoring were nearly two times more likely to be satisfied with their jobs than their counterparts who did not (OR = 2.03; 95%CI: 1.58–1) (Fig. 4).

**Fig. 5:**
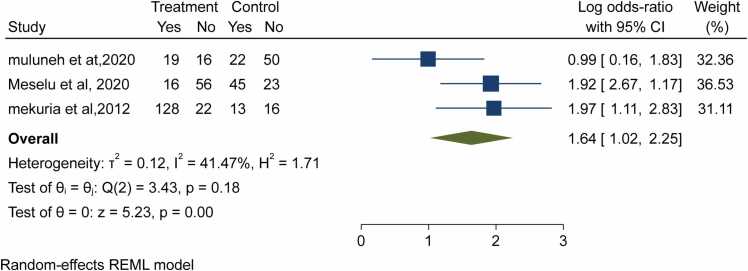
- Forest plot for the association between motivation and job satisfaction among midwives in Ethiopia, 2023.

Motivation was assessed in three studies; it was shown that the probability of being satisfied with their job among midwives with no motivation was high to do their job. The overall estimates revealed that the likelihood of satisfaction were 64% more likely among motivated midwives as compared to those not motivated (OR = 1.64; 95%CI: 1.02–2.25) (Fig. 5).

**Fig. 6:**
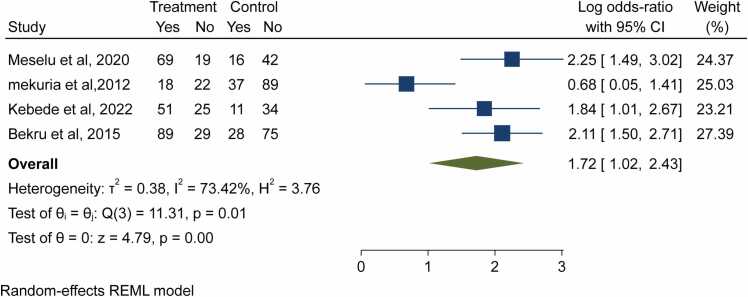
- Forest plot for the association between workload and job satisfaction among midwives in Ethiopia, 2023.

As shown in (Fig. 6), several studies described the about workload related; four studies reported there is positive association between the workload and related factors, and midwives’ job satisfaction, while in the other one study showed there is negative significant association with job satisfaction. Therefore, the overall pooled estimate showed that midwives who reported having a fair workload and encourageable teamwork were more likely to be satisfied with their jobs than their counterparts, who had an excessive workload (OR = 1.72; 95%CI: 1.102–2.43).

**Fig. 7:**
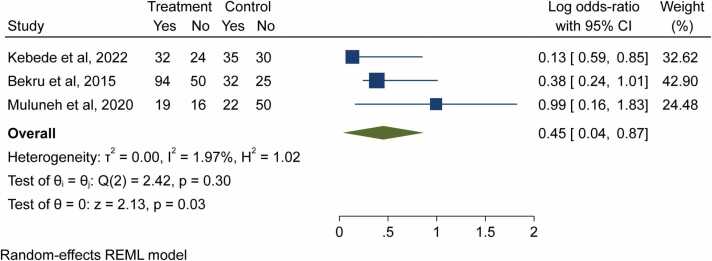
- Forest plot for the association between gender and job satisfaction among midwives in Ethiopia, 2023.

Finally, the effect of sex (being male or female) on the job satisfaction was assessed using 3 studies. One study reported that being female was positively correlated with job satisfaction [11], but two other studies found that male midwives were more likely to be content with their jobs [10], [21]. The pooled analysis demonstrated male respondents were about 55% times less likely to be satisfied than female respondents (OR = 0.45; 95% CI: 0.04, 0.87) (Fig. 7).

## Discussion

This systematic review and meta-analysis mapped out the assessment of factors affecting job satisfaction among midwifery professionals in Ethiopia. The review highlighted the distribution, design, quality and characteristics of the studies. The review included nine studies from different regions which all studies’ analysis used primary data. All of the included studies were institutional-based studies. Three studies included in this review had moderate risk, because of, either lacking information on selection of the sample population or because a sample size not justified.

Despite the absence of systematic reviews and meta-analyses undertaken in Ethiopia or in Africa, we contrasted the current pooled level of job satisfaction among midwives with a number of primary research conducted abroad. The pooled prevalence of job satisfaction of midwifery professionals in Ethiopia was 52.2% with (95% CI =41.7, 62.9). Even though there were no similar studies for this systematic review, the finding was consistent with a systematic review conducted in Iran (46.2%) [27]. This study finding was higher than the systematic review done in Ethiopia among nurse’s professionals which was 41.17% [28]. This poor health professional satisfaction might be due to the safety of the working climate, workload, long working hours, and the leadership in the unit. Besides, these poor motivational factors including incentives and salaries, working climate, and working collaboration were poor in developing countries including Ethiopia [29].

In our sub-group analysis by region revealed that the highest level of midwifery professionals’ job satisfaction was reported in Oromia regional state and the lowest was reported in Amhara regional state of Ethiopia. This may be justified by the rise in patient volume in populated areas, which causes workers to spend more time on duties that degrade care quality and decrease job satisfaction. This supported by meta-analysis conducted on nurses in Ethiopia [28].

In this systematic review and meta-analysis, we found several factors that had an association with level of job satisfaction among midwives in Ethiopia, and that there are similarities and differences between regions in factors affecting it. The present review indicated that socio-demographic and institutional factors play a significant role in job satisfaction. The meta-analysis demonstrated that having satisfaction with their job was positively associated with respondent’s sex, fair supervision, motivation and workload at facilities.

This meta-analysis revealed that fairness of job supervision had significantly associated with job satisfaction. Those who agreed with fairness of supervision were about two times more likely to be satisfied than their counterparts. This finding is supported by a primary study conducted in Canada [30]. This could be because supervision creates a space for frequent conversation, problem-solving, and improved teamwork, which encourages a more supportive, caring, and happy work the environment.

Furthermore, this study found that gender of respondents has positive association with midwives job satisfaction as male respondents were about 55% times less likely to be satisfied than female respondents. This was congruent with a study conducted in USA [31]. This gap may be caused by cultural perceptions of the nature of the job, particularly for those the presumption that midwifery is largely a female profession, and working in labor wards. The literal translation of the term "midwife" in old English as "with women" has also been described in literature, which may offend certain male professionals. The same was also observed with Nigerian nurses [32].

An individual's intensity, direction, and persistence of effort toward achieving a goal can all be attributed to certain processes, which are referred to as motivation. Most of the time, motivation made from a need that must be met, which then prompts a particular behavior: The satisfaction of requirements leads to some kind of reward, which may be intrinsic or external [33]. This finding revealed that the likelihood of satisfaction was more likely among motivated midwives as compared to those not motivated.This finding was supported by study conducted in Cyprus on nursing motivation [34].

Additionally, this meta-analysis showed that midwives who reported having a fair workload and good team work involvement were more likely to be satisfied with their jobs than their counterparts, who had an excessive workload. Participants’ involvement in decision-making and team work processes on work related issues in the health faculties is important to health workers job satisfaction. Workload affects the quality of the provided nursing care by affecting implicit rationing of nursing care, job satisfaction and emotional exhaustion [35]. This finding was supported by systematic review study conducted in sub Saharan countries [36].

## Limitation

Some of the included Studies were conducted on a small number of individuals, which increases the likelihood that the analysis may be biased. Lack of longitudinal studies was one of the major gaps found in this analysis. Furthermore, this systematic review and meta-analysis was not registered with PROSPERO.

## Conclusion

Evidence suggested that in order to keep and be satisfied with health workers at the facilities in Ethiopia, tactics including motivation, fair supervision and job distribution, and building strong relationships with supervisors are crucial. The administrator of midwives in medical facilities needs to recognize the significance of midwifery care quality and the elements that affect it. Regular monitoring of these elements and the implementation of the finest pertinent tactics will ultimately improve the standard of midwifery care, hence boosting job satisfaction.

## Ethical approval and consent to participate

Not applicable; since we did not use primary data that needs ethical approval and consent to participate.

## Funding

No fund received for this study.

## CRediT authorship contribution statement

**Degefa Bekem Dibaba:** Conceptualization, Data curation, Funding acquisition, Supervision, Visualization, Writing – review & editing. **Feyisa Gizu Tola:** Data curation, Formal analysis, Project administration, Software, Validation. **Dinagde Dagne Deresa:** Conceptualization, Data curation, Investigation, Methodology, Software, Supervision, Writing – original draft.

## Declaration of Competing Interest

Authors declared no conflict of interest.
